# Effects of Season, Plumage Colour, and Transport Distance on Body Weight Loss, Dead-on-Arrival, and Reject Rate in Commercial End-of-Lay Hens

**DOI:** 10.3390/ani11061827

**Published:** 2021-06-18

**Authors:** Enver Çavuşoğlu, Metin Petek

**Affiliations:** Department of Animal Science, Faculty of Veterinary Medicine, Bursa Uludag University, Bursa 16059, Turkey; petek@uludag.edu.tr

**Keywords:** end-of-lay hens, transport, distance, season, plumage colour, welfare

## Abstract

**Simple Summary:**

The transport conditions of end-of-lay hens to slaughterhouses are essential for their welfare. In this study, the effect of season, plumage colour, and transport distance on body weight loss, dead-on-arrival rates, and reject rates was examined. Transport-related data of 31.6 million end-of-lay hens over a two-year period from one slaughter plant in Turkey were analysed. Hens transported in the winter and spring seasons had a greater body weight loss and reject rates. Brown-feathered hens had a higher death rate, while white-feathered hens had a higher body weight loss and reject rate. Hens transported longer distances for slaughter had increased body weight loss, death rates, and reject rates. These results indicate that more care should be taken when transporting end-of-lay hens in cold weather and over longer transport distances. We suggest the transport should be reduced to a certain distance, and improved conditions ought to be provided to mitigate losses.

**Abstract:**

Transport conditions of end-of-lay hens are important for their welfare. This study investigated the effects of season, plumage colour, and transportation distance on the welfare of end-of-lay hens. Retrospective data from 31,667,274 end-of-lay hens transported to a poultry slaughterhouse in Turkey were analysed. The mean body weight loss, dead-on-arrival (DOA) rate, and reject rate were 3.723%, 1.397%, and 0.616%, respectively. The effects of season, plumage colour, and transport distance on the evaluated parameters were all statistically significant (*p* < 0.001). The highest body weight loss was found in winter, while the lowest body weight loss was found in autumn. The average DOA rate was highest in spring and lowest in autumn. The highest average reject rate was found in spring (0.630%). Body weight loss, DOA rates, and reject rates were also significantly different among white and brown hens (*p* < 0.001; *p* < 0.001; *p* = 0.016, respectively). The highest body weight loss and reject rates were found in white plumage hens, while the highest DOA rate was found in brown plumage hens. The body weight loss and DOA rate were positively correlated with transportation distance (*p* < 0.001). The results of this study indicate that more preventive measures should be taken during the transport of end-of-lay hens, especially in cold seasons such as winter, and over longer transport distances, in regard to the welfare of these animals. Additionally, the transport of these animals should be lessened to a certain distance.

## 1. Introduction

The numbers of poultry handled, transported, and slaughtered are greater than those for any other livestock. This is because poultry has a shorter generation interval and a better feed conversion ratio compared to other farm animals. Since mass production results in infringement on animal welfare standards, there are many procedures in the poultry production chain being criticised in terms of animal welfare [1]. Apart from raising conditions, poultry transportation is one of the most critical steps in the life cycle of a chicken [2].

Though there are differences between countries and specific production chains, the most common practice is that layer hens are raised in rearing farms up to 16 weeks of age, where they are called pullets at that time [3]. After that, they are transferred to laying farms. On the laying farms, these hens are raised for egg production until the end of their economic life, which depends on several factors such as their egg production performance, egg and feed prices in the market, and the price of a 16-week-old pullet. At the end of their economic life, their market price is determined primarily by their body weight. While end-of-lay chickens are killed on-farm in some countries [4], globally, most of them are transported to and slaughtered in a poultry abattoir [5]. In general, the income from the meat or live bird of end-of-lay hens can be less than the cost of carcass processing [4].

Transport systems need to be designed and managed to ensure poultry are not caused unnecessary distress or discomfort. However, in reality, poultry are exposed to a number of stressors, including thermal stress and long journeys [6]. Thermal stress, either prolonged heat or cold stress, will have detrimental effects on poultry welfare during transportation. Some welfare standards specify that the time between loading the last hen and the time of arrival at the final destination must be less than 8 h [7]. The numbers of birds loaded per drawer according to bird conditions and the weather, lairage time, and environmental conditions are critical factors in animal welfare, from catching to the slaughter of end-of-lay hens. The transportation conditions of poultry, such as the stocking density in the vehicle [8], distance and duration of transportation, climatic condition in the cages, and outside environment, are usually neglected [9]. Additionally, injuries related to handling and loading processes and not providing feed and water during transport increase stress and transport-related mortality [10]. Additionally, sudden speed-ups and breaks of the transport vehicle may also make transportation more stressful for chickens [11]. Due to the low profit margin from the sale of end-of-lay hens, there are not many companies buying and slaughtering these animals. That is why they usually travel longer journeys than other poultry species for slaughter [12].

There are lots of factors affecting layer welfare from catching to slaughter, including catching and transport duration, transport distance, and environmental conditions [7]. Many studies in the literature focus on the effect of transportation on the mortality rate of broiler chickens [13,14]. However, few studies assess the mortality rate of end-of-lay hens during transport. Vecerkova et al. [5] examined 17.4 million layer hens slaughtered in the Czech Republic between 2010 and 2017 and found the average mortality rate to be 0.516%. Additionally, the same study found that the highest mortality rate was found in the cold months (0.717% in January and 0.695% in December), and the lowest rate was found in August (0.364%); longer transportation distances increase the mortality rate significantly. Another study [15] analysed data of the same country (the Czech Republic) from the years 1997–2006 and found the dead-on-arrival rate (DOA) to be 1.013% during the transport of hens and cockerels. De Martino et al. [16] analysed the data of 21.7 million hens transported for slaughter between 2010 and 2012 in Italy and found the median value of DOA to be 0.38%. Furthermore, the same study found that the rate of DOA increases in the winter season; with longer transportation distances, brown-feathered birds had a higher rate of DOA. Weeks et al. [12] analysed the data of 13.3 million hens transported in the UK in 2009 and found the average mortality rate to be 0.27%. It was also found in the same study that the travel distance, external climate conditions, feather cover of the hens, body weight, and poor health of the flock were related to the overall mortality rate. Petracci et al. [9] analysed the data of 19 slaughterhouses of end-of-lay hens between August 2001 and July 2005 in Italy (54 million end-of-lay hens) and found the mortality rate to be 1.22%. Moreover, the same study concluded that the mortality rate was higher (1.65%) in the summer season in Italy. It can be inferred from previous studies that longer travel distances and extreme weather conditions had a negative effect on end-of-lay hens’ welfare.

Almost more than 100 million end-of-lay hens are slaughtered in Turkey every year [17]. Thus far, around 80% of layer hens in Turkey were housed in conventional cages, and 20% of layers were kept in cage-free (free-range or organic) houses. Nevertheless, according to the Turkish Legislation for Protection of Laying Hens [18], conventional cages are planned to be banned in 2023 in Turkey. A total of 70% of all layers are white-feathered in terms of plumage colour, and 30% are brown-feathered, in Turkey. Until now, no study has analysed the mortality rate of end-of-lay hens during the transport to slaughter and factors affecting the mortality rate in Turkey. Additionally, Turkey has a particular climate condition in which hot summers and cold winters can be experienced at the same time in different regions.

This study aimed to investigate the effects of the season of the year, plumage colour, and transport distance on the DOA rate, body weight loss, and carcass reject rate of end-of-lay hens during transport from the farm to the slaughterhouse.

## 2. Materials and Methods

The data of this study were collected from the records of a commercial poultry slaughterhouse in Turkey. The recorded data are from January 2019 to December 2020 of end-of-lay hens transported from almost every region of Turkey. Overall, 31,667,274 hens were transported to this slaughterhouse during this 24-month period. Of all transported hens, consistent with the facts of Turkey’s layer population, 2,337,539 were brown-feathered genotypes, and 29,329,735 were white-feathered end-of-lay hens. To test the effect of plumage colour on each evaluated parameter, each truck was classified according to the plumage colour of the flock transported. The transport of all animals was carried out according to Turkish legislation about farm animals and poultry [19].

The data were categorised into months and years to evaluate the effect of season on DOA, body weight loss, and reject rates. December, January, and February were counted as winter; March, April, and May were counted as spring; June, July, and August were counted as summer; September, October, and November were counted as autumn. The monthly average temperature and humidity recorded at the nearest meteorological station to the slaughterhouse are presented in Table 1.

In order to assess the effect of transport distance, shipments were divided into five subgroups according to lengths travelled: up to 200 km, 201 to 400 km, 401 to 600 km, 601 to 800 km, and 801 km and higher.

When the hens were loaded onto trucks at depopulation, they were caught manually by the company’s staff. Curtains (side sheets) and solid roofs existed in all trucks, but the curtains were used only during cold weather. Ventilation was adjusted by opening the side sheets. Furthermore, each truck consisted of two blocks of crates, and there was a corridor running throughout the length of the vehicle between the two blocks, which was also used for ventilation. Although they varied according to the vehicle, each block consisted of 12 crates in rows and 12 crates in columns, and the crates were fixed or mobile. The average stocking rate in each crate was between 30 and 35 hens depending on weather conditions and the body weight of hens (275–320 cm^2^ per bird).

The DOA rate was assessed as a percentage of the number of animals that died from the catching to shackle process (the process of hanging chickens upside down for slaughter) from the total number of animals transported for slaughter. The number of dead animals was counted during the shackling process by the company’s staff and recorded. Therefore, the DOA rate includes the number of animals that died during transport and the lairage time. This rate was calculated per load of the truck.

DOA rate = number of animals died/number of animals transported × 100

Body weight loss is the body weight loss of animals during transport and lairage. The body weight loss was calculated as a percentage of weight loss of animals from the beginning of the transport to the end of the lairage. Each truck was weighed at the laying farm before (empty weighing) and after loading the hens (full weighing), and the truck was weighed again before being unloaded at the slaughterhouse. The weight difference between the two full weighings was recorded as the body weight loss.

Body weight loss = (Full weight at the farm − Full weight at the slaughterhouse)/Full weight at slaughter) × 100.

The rate of rejected animals was calculated as a percentage of the total number of live animals that reached the slaughterhouse. The rejected animals were those who were alive at the slaughterhouse, but their carcass was not accepted because of anatomical or morphological deformities such as hematomas, emaciated carcasses, broken legs or wings, and wounds or lesions on the body.

Reject rate = (Number of live animals slaughtered − Number of carcasses rejected)/number of live animals slaughtered × 100

The average body weight was calculated as an average value per truck. The calculation was conducted based on the weight of each truck at the exit from the farm.

All data were analysed using the SPSS 23.0 statistical program [20]. Since all data were not normally distributed (tested by the Shapiro–Wilk test), nonparametric tests were used. The Kruskal–Wallis test was used when more than two groups were compared, and the Mann–Whitney U test was used when two groups were compared. For multiple comparisons, Bonferroni correction was used. In order to test the correlations between the variables, the Spearman correlation test was used. A linear (stepwise) regression model was applied for regression analysis. Genotype, season, and distance were used as independent variables.

Significance was stated when *p* ≤ 0.05.

## 3. Results

The average body weight, body weight loss, DOA rate, and reject rate, according to the seasons, are presented in Table 2. All four variables were significantly affected by season (*p* < 0.001). The lowest body weight of hens was observed in the winter season (1.591 kg), while the highest body weight of hens was observed in the autumn season (1.625 kg). The highest body weight loss was detected in the winter season (4.020%), while the lowest body weight loss was observed in the autumn season (3.462%). In terms of the DOA rate due to transport, the highest rate was seen in the spring season (1.590%), while the lowest rate of mortality was seen in the autumn season (1.077%). The percentage of rejected animals at slaughter was highest in the spring season (0.630%).

The average body weight, body weight loss, DOA rate, and reject rate, according to plumage colour, are presented in Table 3. All four parameters were significantly different in plumage colour (*p* < 0.001). The average body weight of brown layers (1.853 kg) was higher than white layers (1.586 kg). The average body weight loss and reject rate were higher in white layers (3.772%; 0.617%) than brown layers (3.178%; 0.607%). On the other hand, the average DOA rate was higher in brown layers (1.644%) than in white layers (1.375%).

The average body weight, body weight loss, DOA rate, and reject rate, according to transportation distances between the farms and the slaughterhouse, are presented in Table 4. Each evaluated variable was significantly affected by the transport distance (*p* < 0.001). The body weight loss, DOA rate, and reject rate were lower for shorter transportation distances, while all the evaluated parameters were higher when the transportation distance increased.

Transport distance and body weight were tested for correlations with body weight loss, mortality rate, and reject rate, and the results are presented in Table 5. Transport distance was positively correlated with body weight loss and the DOA rate (*p* < 0.001). Average body weight was negatively correlated with body weight loss and the reject rate (*p* < 0.001) and positively correlated with the DOA rate (*p* < 0.001).

**Figure 1:**
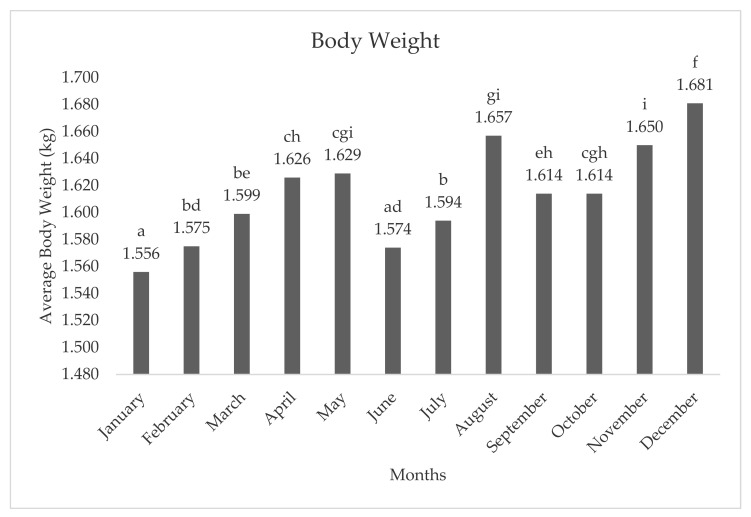
Average body weight at the end of lairage of hens by month. **a**–**f**; different letters indicate statistical differences.

The average body weight of hens in each month is presented in Figure 1. The lowest body weight of hens was seen in January (1.556 kg), while the highest body weight was seen in December (1.681 kg).

Genotype, genotype x distance, and distance x genotype x season were found to be significant in predicting body weight loss (*p* < 0.001) (Table 6). Distance and distance x season were significant, whereas genotype was not significant, within the model, in predicting the DOA rate. The model for predicting the reject rate was not significant for all variables (*p*-value = 0.983). The reject rate could not be predicted by genotype, season, and distance.

**Figure 2:**
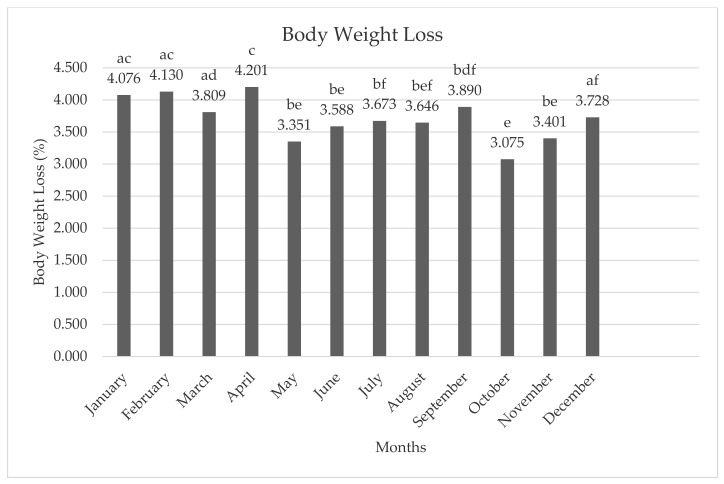
The average body weight loss of hens according to months. **a**–**f**; different letters indicate statistical differences.

The average body weight loss due to the transport of hens to the slaughterhouse is presented in Figure 2. The lowest body weight loss was seen in October (3.075%), while the highest rate was seen in April (4.201%).

**Figure 3:**
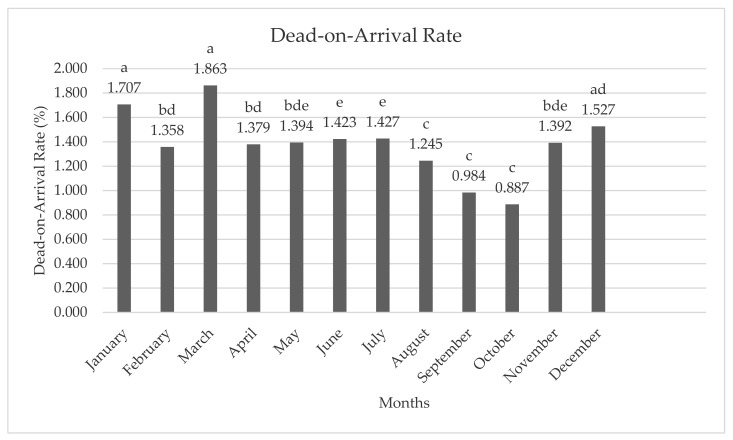
Average dead-on-arrival rate of hens according to months. **a**–**d**; different letters indicate statistical differences.

The DOA rate of hens in each month is presented in Figure 3. The lowest DOA rate was seen in September (0.984%) and October (0.887%), while the highest DOA rate was seen in March (1.863%).

**Figure 4:**
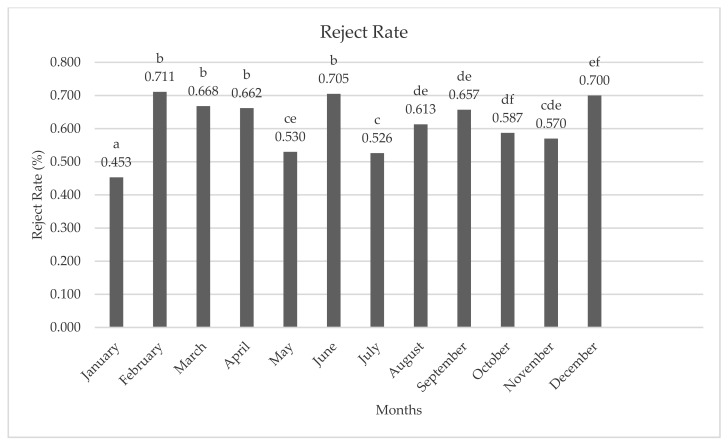
Average reject rate of hens according to months. **a**–**f**; different letters indicate statistical differences.

The average reject rate of hens slaughtered in each month is presented in Figure 4. The average reject rate was the lowest in January (0.453%), while the highest reject rate was seen in February (0.711%) and June (0.705%).

## 4. Discussion

Pre-slaughter handling in poultry production is potentially essential for animal welfare implications, including hen mortality. In order to reduce the percentage of DOA, specific risk factors need to be identified, and preventive measures should be taken. The DOA rate and body weight loss of broilers and other meat-producing ruminants are critically analysed, and careful precautions are taken since the primary income from these animals is their meat production. However, the economic value of laying hens become very low when they complete their productive life since their carcass weight and meat price is meager compared to broilers and turkeys. That is why the welfare of end-of-lay hens is not critically evaluated by producers or retailers. However, it is an ethical and legal responsibility to maintain the welfare of all animals at a certain level. Freedom from pain and hunger is one of the five pillars of the freedom of animals [7]. Therefore, it is crucial to identify the risk factors related to the DOA rate and body weight loss of end-of-lay hens during their depopulation and transport.

The average DOA rate within the evaluated period was 1.397%. Since there are around 100 million laying hens in Turkey [17], this mortality rate during the transport to slaughter causes a significant economic loss to the laying hen industry. There were 58.1 million boiling fowl (end-of-lay hens and end-of-lay breeders) slaughtered in the UK in 2020 [21]. The number of commercial laying hens in the United States in December 2019 was 340 million [22]. The numbers also show that the transport of end-of-lay hens is a great economic issue for many countries, apart from the animal welfare point. Weeks et al. [12] found the average mortality rate of end-of-lay hens in Great Britain to be 0.27%, while Petracci et al. [9] found the mortality rate of laying hens to be 1.22% in Italy. Vecerkova et al. [5] analysed 17.4 million end-of-lay hens and found the average mortality rate due to transport to be 0.516%. The average mortality rate we found in the current study was slightly higher than in previous studies [5,9,12]. Similarly, the average body weight loss and reject rates were 3.723% and 0.616%. These results also show that transport-related economic loss in laying hen production should be carefully evaluated; critical points should be revealed.

When the body weight loss by seasons was evaluated in our study, it was found that the highest body weight loss was seen in the winter season. Similar to seasons, the highest body weight loss according to months was found in January, February, March, and April. When the birds are transported in the winter seasons, they probably face very cold environmental conditions, and they lose weight in order to sustain their body temperature. Similar to the body weight loss, the highest DOA rate (1.532%) was also seen in the winter and spring seasons. Similar to seasons, the highest DOA rate according to months was found in December, January, and March. The end-of-lay hens in some flocks are mostly poorly feathered because of feather pecking, but also because the beginning of moult or husbandry factors can trigger mechanical wear and tear. A precise assignment of the causes of feather loss is not always possible [23]. Poor feather coverage makes it difficult to control their body temperature in cold ambient conditions. The average ambient temperature in winter seasons may drop down to below 0 degrees Celsius in most of Turkey [24]. That is why cold environmental conditions cause more death than summer seasons. This could be caused by challenging climate conditions in Turkey and may be due to longer transport distances. Previous studies also found a higher mortality rate in cold seasons [5,12,16,25].

The reasons for rejection of the carcasses of end-of-lay hens in slaughterhouses are pre-slaughter handling, the slaughter process (mistakes caused by mechanical problems), and problems in the hens’ bodies. The defects in the bodies of animals are hematomas, emaciated carcasses, wounds and lesions in the body, broken legs and/or wings, etc. Similar to the DOA rate, the rejected carcass rates were higher in the spring season among all seasons. Similar to seasons, the highest body weight loss according to months was found in December, February, March, and April. It can be noted that the transport of hens in cold weather conditions causes higher death rates and increases the carcass problems of hens.

Concerning the prevalence of white-feathered/brown-feathered hens among all layers in Turkey, 70% of layers are white-feathered layers. In terms of plumage colour, the main proportion of the hens slaughtered in this plant had a white plumage. The average body weight, according to plumage colour, was higher in brown-feathered hens, as expected. However, the average body weight loss was higher in white-feathered birds. This means that transportation causes more body weight loss in hens with a lower body weight. Hens with a lower body weight might be more affected by transport-related stress. However, hens with brown feathers showed a higher mortality rate due to transport. While the body weight loss was higher in white plumage hens, the DOA rate was higher in brown-feathered hens (Table 3). The average reject rate, according to plumage colour, was found in white-feathered hens.

The DOA rate was higher for longer transport distances. The DOA rate (2.961%) for transport distance V (800 km and more) was almost three times higher (1.027%) than for distance I (0–200 km). Similarly, the average body weight loss was higher for the longer transport distances. Distance also had the highest effect on body weight loss within the regression model (Table 6). This is because longer distances cause more transport-related stress [5,9]. On the other hand, the highest reject rate was found for transport distance III (400–600 km). The negative effect of longer transport distances on poultry welfare was also shown in previous studies. Many previous studies on end-of-lay hens found that longer transportation distances increase the mortality rate of animals [5,9,12,16]. The DOA rate in the current study was higher than in previous studies because the hens in this study were transported longer distances and might have faced harder climate conditions than those of previous studies. Apart from laying hens, the negative effect of longer transportation distances was also seen in other poultry species. Caffrey et al. [14] found a higher mortality rate in for long transport distance of broiler chickens. Similarly, Machovcova et al. [26] found the negative effect of longer transport distances in turkeys. In order to decrease these problems, either these animals should be slaughtered in closer slaughterhouses, or the transport conditions over longer distances should be more strictly monitored; at least, water and feed should be provided for durations longer than 12 h.

In this study, the correlations between different variables were analysed, and it was found that transport distance was positively correlated with body weight loss (*r* = 0.404) and the mortality rate (*r* = 0.294). These results suggest that more careful precautions are needed to be taken when animals are transported for long distances. Furthermore, body weight was negatively correlated with body weight loss (*r* = −0.078) and the reject rate (*r* = −0.182). Hens with a higher body weight had a lower body weight loss and reject rate, meaning that farmers should maintain the body weight of their flock at a certain level in order to avoid the negative effects of transport stress. The findings found in regression models (Table 6) support the hypothesis that transport distance has a higher effect on the DOA rate, body weight loss, and reject rate. On the contrary, body weight was positively correlated with the mortality rate (*r* = 0.078). This result seems to be related to plumage colour since the body weight of brown layers was higher than the white layers, and therefore the mortality rate was higher in brown layers (Table 3). That is why the mortality rate was found to be positively correlated with body weight.

This study, on the other hand, had some limitations. It would be better to know the total time each truck spent during the transport, the number of breaks given, and how the animals were ventilated during the breaks. Secondly, during transportation, if the instantaneous temperature and humidity values of several points of the truck chassis were measured with a data logger, the effect of climatic conditions would be better demonstrated. It is recommended that future studies should also consider these issues to reveal the transport-related problems sufficiently and obtain more reliable findings.

## 5. Conclusions

In conclusion, the transportation of end-of-lay hens is a crucial stage in their life. In general, cold seasons caused more body weight loss and increased mortality. While body weight loss and the reject rate were higher in white-feathered hens, mortality was higher in brown-feathered hens. When hens were transported longer distances, the negative effect of transport stress, such as the mortality rate and body weight loss, increased. These results also suggest that more consideration should be taken during the transport of these animals, especially in cold weather and over longer transport distances. The internal and external temperatures of crates may be monitored instantly, and extra precautions can be taken if needed in order to protect hens from extreme weather conditions.

## Figures and Tables

**Table 1:** Average temperature and humidity in 2019–2020 in the nearby region of the slaughterhouse.

	January	February	March	April	May	June	July	August	September	October	November	December
Temp. °C	3.55	5.71	8.70	11.54	17.57	20.98	22.67	22.88	20.58	16.23	9.33	6.93
Humid.%	82.05	79.03	71.52	68.39	70.14	73.34	68.76	66.14	68.02	75.17	79.05	79.81

**Table 2:** Average body weight, body weight loss, dead-on-arrival rate, and reject rate according to seasons (mean ± SEM).

Season	Number of Trucks	Body Weight (kg)	Body Weight Loss (%)	Dead-On-Arrival Rate (%)	Reject Rate (%)
Winter	696	1.591 ± 0.004 ^c^	4.020 ± 0.074 ^a^	1.532 ± 0.066 ^a^	0.608 ± 0.034 ^c^
Spring	858	1.615 ± 0.004 ^b^	3.812 ± 0.059 ^a^	1.590 ± 0.093 ^a^	0.630 ± 0.009 ^a^
Summer	935	1.600 ± 0.004 ^c^	3.633 ± 0.083 ^b^	1.384 ± 0.110 ^b^	0.619 ± 0.011 ^b^
Autumn	767	1.625 ± 0.004 ^a^	3.462 ± 0.072 ^b^	1.077 ± 0.053 ^b^	0.606 ± 0.012 ^b^
Average	818.5	1.608 ± 0.002	3.723 ± 0.037	1.397 ± 0.044	0.616 ± 0.009
*p*-value		<0.001	<0.001	<0.001	<0.001

^a–d^ Different superscripts in columns indicate statistical significance.

**Table 3:** Average body weight, body weight loss, dead-on-arrival rate, and reject rate according to plumage colour (mean ± SEM).

Plumage Colour	Number of Trucks	Body Weight (kg)	Body Weight Loss (%)	Dead-On-Arrival Rate (%)	Reject Rate (%)
White Plumage	3001	1.586 ± 0.001	3.772 ± 0.039	1.375 ± 0.047	0.617 ± 0.009
Brown Plumage	273	1.853 ± 0.007	3.178 ± 0.113	1.644 ± 0.143	0.607 ± 0.024
*p*-value		<0.001	<0.001	<0.001	0.016

**Table 4:** Average body weight, body weight loss, dead-on-arrival rate, and reject rate according to transportation distance (mean ± SEM).

Distance (km)	Number of Trucks	Body Weight (kg)	Body Weight Loss (%)	Dead-On-Arrival Rate (%)	Reject Rate (%)
I (0–200)	538	1.609 ± 0.005 ^a^	3.038 ± 0.099 ^a^	1.027 ± 0.084 ^a^	0.648 ± 0.044 ^a^
II (201–400)	1390	1.624 ± 0.003 ^b^	3.047 ± 0.043 ^a^	1.131 ± 0.073 ^a^	0.590 ± 0.008 ^a^
III (401–600)	692	1.579 ± 0.004 ^c^	4.043 ± 0.064 ^b^	1.408 ± 0.071 ^b^	0.649 ± 0.013 ^b^
IV (601–800)	384	1.634 ± 0.006 ^b^	4.280 ± 0.077 ^c^	1.770 ± 0.098 ^c^	0.590 ± 0.016 ^a^
V (801- above)	268	1.561 ± 0.004 ^c^	6.841 ± 0.154 ^d^	2.961 ± 0.243 ^d^	0.641 ± 0.020 ^b^
*p*-value		<0.001	<0.001	<0.001	<0.001

^a–d^ Different superscripts in columns indicate statistical significance.

**Table 5:** Spearman correlation coefficients between different variables (*r* (*p*-value)).

	Body Weight Loss	Mortality Rate	Reject Rate
Transport Distance	0.404 (<0.001)	0.294 (<0.001)	0.033 (0.062)
Body Weight	−0.078 (<0.001)	0.078 (<0.001)	−0.182 (<0.001)

**Table 6:** Linear regression analysis between different variables.

Parameter	Models		Model Adjusted R Square	Regression *p*-Value	Coefficients St. Beta	
					St. Beta	Significance
Body Weight Loss	1	Distance	0.203	<0.001	0.451	<0.001
	2	Distance	0.209	<0.001	0.452	<0.001
		Genotype			−0.082	<0.001
	3	Distance	0.212	<0.001	0.448	<0.001
		Genotype			−0.079	<0.001
		Season			−0.058	<0.001
Dead-On-Arrival	1	Distance	0.033	<0.001	0.182	<0.001
	2	Distance	0.035	<0.001	0.178	<0.001
		Season			−0.054	0.002
